# Mixed-Methods Research in a Complex Multisite VA Health Services Study: Variations in the Implementation and Characteristics of Chiropractic Services in VA

**DOI:** 10.1155/2013/701280

**Published:** 2013-12-31

**Authors:** Raheleh Khorsan, Angela B. Cohen, Anthony J. Lisi, Monica M. Smith, Deborah Delevan, Courtney Armstrong, Brian S. Mittman

**Affiliations:** ^1^VA Center for Implementation Practice and Research Support, VA Greater Los Angeles Healthcare System, 16111 Plummer Street, Sepulveda, Los Angeles, CA 91343, USA; ^2^Military Medical Research Program, Samueli Institute, Corona del Mar, CA, USA; ^3^Chiropractic Program, Patient Care Services, Veterans Health Administration, Washington, DC, USA; ^4^Chiropractic Service, VA Connecticut Healthcare System, West Haven, CT, USA; ^5^RAND Corporation, Boston, MA, USA

## Abstract

Maximizing the quality and benefits of newly established chiropractic services represents an important policy and practice goal for the US Department of Veterans Affairs' healthcare system. Understanding the implementation process and characteristics of new chiropractic clinics and the determinants and consequences of these processes and characteristics is a critical first step in guiding quality improvement. This paper reports insights and lessons learned regarding the successful application of mixed methods research approaches—insights derived from a study of chiropractic clinic implementation and characteristics, Variations in the Implementation and Characteristics of Chiropractic Services in VA (VICCS). Challenges and solutions are presented in areas ranging from selection and recruitment of sites and participants to the collection and analysis of varied data sources. The VICCS study illustrates the importance of several factors in successful mixed-methods approaches, including (1) the importance of a formal, fully developed logic model to identify and link data sources, variables, and outcomes of interest to the study's analysis plan and its data collection instruments and codebook and (2) ensuring that data collection methods, including mixed-methods, match study aims. Overall, successful application of a mixed-methods approach requires careful planning, frequent trade-offs, and complex coding and analysis.

## 1. Introduction

There is growing consumer interest in complementary and alternative medicine (CAM) in the USA and internationally [1–3]. Healthcare systems have responded to this demand by offering a range of CAM services in outpatient and inpatient settings [4, 5]. Patients enrolled in the US Department of Veterans Affairs (VA) healthcare delivery system often use CAM services outside of VA but have a strong interest in receiving these services within the VA system [6–11]. In response, VA began providing selected in-house CAM services in about 2001 [12]. VA's most substantial undertaking in delivering any CAM-related service has been its introduction of chiropractic services.

Chiropractic care is often described as sitting at the crossroads of CAM and mainstream medicine [13], and its introduction into the VA healthcare system exemplifies that duality. In 1999, Congress directed VA to establish a policy regarding chiropractic services for musculoskeletal conditions (Public Law 106–117) [14]. Although specific action was not mandated, in response to this legislation, VA began providing limited access to chiropractic care by paying for services delivered outside the VA healthcare system. In 2001, Public Law 107–135 made chiropractic services part of the standard medical benefits available to all Veterans and required VA to deliver these services on-site by VA chiropractors at a minimum of one VA medical facility in each of VA's 21 geographic regions (Veterans Integrated Service Networks or VISNs) [14, 15]. This required the incorporation of a new provider type, doctors of chiropractic (DCs), into VA's clinical and administrative policies and procedures.

The establishment of chiropractic clinics within VA was challenged by the rarity of existing models in other healthcare systems and by the widely varying perception of chiropractic services by medical physicians and other stakeholders [16]. VA convened a Federal Advisory Committee to make recommendations on the implementation of chiropractic services and in July 2004 issued Directive 2004–035 which established the overall policy for VA chiropractic services. While chiropractic care is now part of VA's standard medical services, in practice and perception chiropractic care still retains many of the limiting features of a CAM service within a traditional medical setting. The introduction of chiropractic services in VA faced not only the typical challenges of introducing any new clinical service or program into a large healthcare system but also the unique obstacle of integrating a nontraditional healthcare service into conventional medical settings [14, 17–19].

By the end of 2005, VA had successfully complied with the requirement of establishing a minimum of one chiropractic clinic within each VISN. This initiative was loosely coordinated by VA Central Office (VACO), leaving much of the details to individual facilities. Over the following years, the use of chiropractic services at these initial facilities dramatically increased. This growth, along with interest from Veterans and providers at other VA facilities, stimulated the expansion of chiropractic clinics into other VA facilities. From fiscal year 2005 to fiscal year 2011, without further Congressional mandate, the number of VA chiropractic clinics increased from 24 to 43, and the number of Veterans receiving care at these clinics increased from just under 4,000 to over 81,000. Also during this time, VACO established central leadership for the chiropractic program in the Office of Rehabilitation Services which began to monitor and assess the ongoing uptake and expansion of services [20]. Because of expected challenges facing the introduction of a new provider type (issues of privileging, competencies, and facility integration), unique features related to chiropractic care (varying perception and prior experience of other clinicians), and the relatively decentralized manner in which initial clinics were established, the chiropractic program office sought to gain deeper knowledge of the program's continuing development and features.

Early studies of chiropractic care in VA described patient characteristics and outcomes in individual VA chiropractic clinics [17, 21–23], characteristics of patients and clinics at the national level [14], and elements of academic training programs [24]. However, a more in-depth understanding of VA's implementation of chiropractic services was needed to inform future policy and practice decisions and ultimately to ensure the highest quality of care delivered to Veterans. Program implementation initiatives within VA, as well as similar efforts outside VA, require careful planning and execution to achieve success. The chiropractic program office lacked the resources and expertise to conduct a large-scale program evaluation but was positioned to build partnerships with the VA research community. These circumstances led to a research-policy-practice partnership established to design and obtain funding for a program of research, beginning with a pilot study entitled “Variations in the Implementation and Characteristics of Chiropractic Services in VA (VICCS).” The VICCS study was guided by prior research examining the introduction and integration of nurse practitioners in VA [25] and related research examining the introduction and role of nurse practitioners and physician assistants in other healthcare delivery settings [26], as well as additional studies documenting the implementation and integration of new clinical services in a range of settings.

The VICCS research-policy-practice partnership sought to explore the chiropractic services program in parallel with other VA integrated care initiatives. These include programs for Veterans returning from operations in Afghanistan and Iraq (i.e., VA's *Post-Deployment Integrated Care Initiative*) as well as a national palliative care program (*Comprehensive End-of-Life Care Initiative*) and the ongoing primary care medical home initiative (*Patient Aligned Care Teams*) [17].

This paper describes the design and methods of the VICCS study and insights gained from the application of a mixed-methods approach to address study questions. The experiences and insights from the study offer guidance for future research-practice partnerships and methods suitable for assessing the introduction of other new clinical services—traditional or CAM—in VA and other large healthcare systems. The paper describes the mixed-methods design employed, as well as specific challenges and issues related to data collection instruments and data collection logistics, analyses of diverse data types for distinct study aims, and other issues.

## 2. VICCS Study Development and Aims

The primary objective of the VICCS study was to identify variations in the implementation processes and organizational arrangements of VA chiropractic services and examine the causes and consequences of those variations. A mixed-methods approach was used to pursue the study's three specific aims.

1. Document and characterize (a) the implementation of chiropractic services into individual VA healthcare delivery facilities and (b) the characteristics and organizational arrangements through which these services are delivered, including their integration with existing clinical services.
2. Identify (a) key factors leading to different implementation patterns and clinic characteristics across different VA facilities and (b) selected impacts and consequences of different implementation patterns and clinic characteristics.
3. Develop and refine research methods and tools for (a) a larger, more definitive study of chiropractic care programs in VA and for (b) studies examining the implementation of other new services and disciplines (including CAM services) in large healthcare delivery systems.

To address VICCS study aims (1) and (2), the study employed a comparative case study approach relying on (a) interviews to gather data from key stakeholders, (b) collection and content analysis of policy and procedure documents and other archival/documentary material to supplement interview-provided data, and (c) administrative data on use of VA chiropractic services. The study team's experience and identification of several methodological and logistical challenges encountered during the study contribute to VICCS study aim (3), in which the study team used many of the “lessons learned” to inform and guide planning for future studies, whether of chiropractic services in VA or other new services and disciplines in any large US healthcare delivery system.

The mixed-methods approach included qualitative and quantitative analysis methods for inductive (hypothesis generation and exploration) and deductive (hypothesis testing) analyses. VICCS study data collection occurred in 2010 and 2011, followed by data analysis and reporting in 2012.

## 3. Mixed Methodology and Health Services Research

The VICCS study's core conceptual framework relied on Donabedian's structure, process, and outcomes model (Table 1) [27]. Donabedian [28] suggests that the quality of health care can be conceptualized and evaluated along three main dimensions of care delivery: structures of care, processes of care, and care outcomes. Structure refers to the setting in which care is delivered, including facilities and equipment, qualification of care providers, administrative structure, and operations of programs. Process encompasses how care is provided and is measured in terms of appropriateness, acceptability, completeness, or competency [29–33]. These measurements are typically less definite than those obtained through assessing outcomes. Lastly, outcomes refer to the end points of care, such as improvement in function, recovery, or survival. Outcomes are usually concrete and precisely measured [34].

The VICCS data collection framework was designed to include several key categories of variables. These included features of each site and the background and motivation for the establishment of each chiropractic clinic. For example, information was collected on the initial impetus for each clinic (i.e., did VISN leadership *require* establishment of a clinic at a given facility or did facility leadership *voluntarily* establish a clinic?) and other key features of the healthcare setting prior to chiropractic clinic implementation. The data collection framework also distinguished several distinct phases in the clinic planning, implementation, and maintenance process and several distinct categories of variables describing the clinic context, chiropractic clinic itself, and key outcomes and measures of performance at each clinic.

Table 1 lists the domains and illustrative variables selected for the VICCS study. The conceptual model was further refined through an iterative process as the study was underway, as described in the following.

- *Environment/context* includes local factors, such as local stakeholder attitudes toward innovation in general and chiropractic in particular, as well as VA regional and national factors, and non-VA external factors such as Veteran Service Organization influences.
- *Planning/implementation* includes features of a facility's planning process and the participation of various stakeholders with differing levels of subject matter expertise.
- *Clinic structure* includes characteristics of the individual DC clinician(s), organizational alignment, and physical features of the clinic, the formal relationship, and extent of integration or collaboration with other facility programs and stakeholders.
- *Care processes *include characteristics of healthcare services provided, features of case management or care pathways, and quality of services.
- *Impacts/outcomes *include the status of clinic access and use, patient-based outcomes, system perception of value, and external stakeholder opinions.

Semistructured interview guides were developed for each type of stakeholder. Interview subjects included VA facility leaders, chiropractors, chiropractor supervisors, chiropractic clinic support staff, other clinicians from various departments, administrative planners, VA patients with musculoskeletal pain complaints (from both chiropractic and nonchiropractic clinics), and external stakeholders (academic affiliates, Veteran Service Organizations, and former Federal Advisory Committee participants). Interview guides were adapted from existing instruments employed in similar studies, augmented by new questions and content specific to this study. Questions to measure stakeholder satisfaction and views were informed by existing instruments such as the Chiropractic Satisfaction Questionnaire [35], the Measure of Clinicians' Orientation toward Integrative Medicine (IM-30) [36], and input from subject matter experts.

## 4. Mixed-Methods Approach 

Mixed-methods research is increasingly used in the social, behavioral, and health sciences. The VICCS mixed-methods approach employed an explicit conceptual framework identifying key variables and data sources relevant to the study's primary aims. This approach improves the quality and completeness of data in health care research to study issues as varied as health disparities, cultural differences, behavioral factors contributing to disability and health, processes and factors involved in implementation of health research findings, and much more. The increasing use of mixed methods reflects growing recognition of the value of qualitative and other social science research methods, collaborative interdisciplinary research teams (also known as team science) [37, 38], and the use of multilevel approaches to investigate complicated health issues [39]. Such approaches are often combined with clinical trials, surveys of attitudes and beliefs, and epidemiological measures to better understand health and illness [40, 41].

Creswell et al. [40] document current trends in the application of mixed-methods approaches in a broad range of health-related research, such as in cardiology [42], pharmacy [43], family medicine [44], pediatric oncology nursing [45], mental health services [46], disabilities [47], and public health [48]. The settings vary from the clinic [49] to the social context of daily activities and relationships [50]. Trends in mixed-methods research are also documented in a study of NIH-funded investigations that incorporated “mixed methods” or “multimethods” in NIH-funded research abstracts [51]. Qualitative methods are used in mixed-methods studies to address broad, open-ended, and interconnected questions that are often quite different from conventional clinical hypotheses [52]. Many social scientists view inductive, interpretive, and related applications of qualitative methods as an important advantage over quantitative methods in developing insights into values, beliefs, attitudes, and interpretations of current or past events and other phenomena, but these methods can be used to supplement quantitative methods to examine many other phenomena as well.

## 5. Results

### 5.1. Sampling, Site and Subject Selection, and Recruitment

The VICCS study data included extensive notes from one hundred eighteen interviews. Most interviews were conducted in person (84%, *n* = 99) during two-day site visits at seven facilities. The remaining interviews were completed by telephone (16%, *n* = 19) with VA facility staff unavailable during the site visit and with external stakeholders. Documents were collected during site visits and received via fax and e-mail prior to and following site visits.

Sampling procedures and criteria were developed for seven study sites. As the VA National Director of chiropractic services, the study's co-PI (AJL) was invaluable given his expertise and experience in administrative and subspecialty matters. However, to avoid potential coercion or bias, AJL did not participate in the site selection decision-making process, recruitment, or interviews with any chiropractic staff (including chiropractors, their supervisors, or support staff).

Sites were selected to ensure diversity on key dimensions, including:

- facility geographic location (regions of USA, urban/suburban/rural);
- facility type (medical center versus outpatient clinic, complexity);
- administrative alignment (facility service line overseeing the clinic);
- chiropractor characteristics (appointment type, full-time versus part-time, clinical experience, prior practice setting, credentials);
- clinic establishment (how long the clinic had been in existence);
- involvement with academic affiliate(s).

At the time of site selection, forty-one (41) VA facilities offered on-site chiropractic services. Two (2) sites involved the study's co-PI as a staff member and were thus excluded. Two (2) other sites were deemed ineligible because they functioned as independent outpatient clinics and were not directly linked administratively to VA. Another site was ineligible because there was no chiropractor on staff at the time of site selection. Therefore, a total of thirty-six (36) sites were eligible for recruitment.

We set a sample size of seven sites, including one pilot site. A total of 12 sites were invited to participate until we reached our sample of seven, for a total site response rate of 58%. Of the five (5) sites that declined, 3 declined due to workload or other time conflicts, and 2 declined because facility leadership determined that local IRB review would be required (based on their perception that their sites would be actively engaged in the research, thereby triggering the need for local IRB review), thus disqualifying them from meeting the study's timeline. Facilities invited but declining to participate in this study remain confidential to those outside of the study team as well as to the study's co-PI, as per the study's recruitment protocol and a confidentiality feature designed to minimize coercion.

At each site, we targeted a variety of stakeholders for interviews:

- facility leadership (facility directors, chiefs of staff);
- key department heads (e.g., primary care, physical medicine and rehabilitation, orthopedics, neurology, pain clinic, rheumatology, radiology, and spine clinic);
- chiropractors;
- clinicians (primary care and specialty providers who both did or did not refer patients to chiropractic service) (2 per discipline, for a total of 6–8 per site);
- chiropractic clinical and administrative support staff;
- VA back or neck pain patients (2-3 from each chiropractic clinic and 2-3 from a nonchiropractic back pain or related clinic, each seen at VA three or more times for the same neck or back issue);
- external stakeholders (local, such as academic affiliates, and national, such as national VSO representatives and federal advisory associates).

Prior to data collection, all study team members attended interview training and observed at least two pilot interviews. Two or three members of the study team attended each site visit, with one or two members conducting each interview. All subjects orally consented prior to participation. For respondents who agreed to be audio-recorded, these interviews were transcribed. Patients were not audio-recorded as per our protocol, but copious notes were taken and debriefing sessions occurred immediately afterwards to ensure that as much verbatim information was retained as possible. Excluding the 18 patients interviewed, 96 of the remaining 100 interview subjects agreed to be audio-recorded. To ensure confidentiality of sites and subjects, all identifiers were removed and replaced with study-generated consecutive identification codes prior to data coding and analysis.

### 5.2. Qualitative and Quantitative Data

Between December 2010 and November 2011, 118 semistructured interviews were conducted at seven sites, including one pilot site. Interview subjects included sixty-two non-DC clinicians (53%), eighteen patients (15%), eleven leaders (9%), seven chiropractors (6%), six chiropractic support staff (5%), five staff involved in planning chiropractic clinics (4%), four chiropractic supervisors (3%), three former federal chiropractic advisory committee members (3%), and two academic affiliates (2%). Participation response rates for subjects and sites were 43% and 58%, respectively.

Documents reviewed and analyzed included significant chiropractic care-related policy documents obtained from the study sites, including VA regional (VISN) policies, local facility policies, local service agreements, chiropractor clinician privileges, and other public documents such as congressional bills/resolutions related to VA chiropractic services.

Content analysis of interviews and documents assessed a priori hypotheses derived from prior literature, as well as new themes emerging from transcript review. The codebook for the interviews and documents was developed and refined throughout the coding process using top-down (deductive/a priori hypothesis testing) and bottom-up (inductive/emerging hypothesis generating) methods.

Data collected from both interviews and documents were coded using NVivo (QSR International) and Excel (Microsoft) software, respectively. We observed high interrater agreement (*k* = 0.8) among coding team members. Data were coded in a two-phased process utilizing high level codes first (double coded) for general themes and variable domains and then more specific detailed codes for subthemes and individual variables.

Additionally, the study obtained quantitative administrative data on clinic use and utilization characteristics such as patient visit counts, patient demographics, diagnoses seen, and services delivered. These data were obtained from the VA Corporate Data Warehouse via VA Informatics and Computing Infrastructure (VINCI).

The VICCS study was approved by four separate institutional review boards (IRBs): VA Greater Los Angeles Healthcare System, VA Connecticut Healthcare System, Western IRB, and US Army Medical Research and Materiel Command.

## 6. Discussion

### 6.1. VICCS Study Methodological Issues and “Lessons Learned”

Most of the methodological challenges in the VICCS study fall into 4 main categories: (1) subject selection and recruitment, (2) site selection, (3) data collection instruments and logistics, and (4) analysis of and interpretation of diverse types of data related to the study's multiple research questions and goals.

#### 6.1.1. Selection and Recruitment of Participants

The site selection process was designed to ensure wide variability of chiropractic clinics and their internal structures and processes. However, because participation of sites and stakeholders at each site was voluntary, some bias may have been introduced that will limit any generalizations outside of VA.

Recruitment of busy healthcare professionals (both clinicians and administrators) is a well-recognized challenge to conducting research in healthcare settings [53]. Because some clinicians and staff are unionized, sites also had to have union notification and, in some cases, approval (local and national).

VA and related federal regulations on patient survey/interview research allow small numbers of patients to be sampled in all IRB-approved studies, but, for studies attempting to recruit larger numbers of patients (>9), additional approval of the Office of Management and Budget is required. Because OMB approval is lengthy, we sampled a smaller number of patients directly and attempted to gather additional patient perspectives indirectly through provider interviews.

#### 6.1.2. Site Selection

In partnership research involving program leaders who serve as research team members, research subjects (i.e., staff from participating sites) may be concerned that they are being evaluated on their performance and actions rather than studied for the purpose of scientific knowledge development. Therefore, issues of possible coercion and sensitivities may affect the validity of data collected.

Staff turnover and limited memories also threaten data validity (temporal bias) when studying program development and evolution in a retrospective manner. Archival records are limited, and thus we relied heavily on VA staff (where still available) and their memories.

To improve the validity and integrity of data collected, this study performed most interviews in person during a two-day site visit (with two to three research team members) at each of the seven sites; 84% of all interviews were conducted in person (*n* = 99). To minimize data security and privacy concerns with IRBs, patients were recruited for interviews onsite and only first names were used.

Two patient respondent groups participated in this study based on their personal experience with three or more visits related to back or neck pain issues: those who received chiropractic services and those who received other nonchiropractic services (e.g., primary care, neurology, and orthopedics). Patients in clinic waiting areas were recruited systematically. No one was missed or avoided. And, because of the study's IRB-approved minimal risk status, this study was granted a waiver of documentation of informed consent; thus all subjects orally consented to participate in this study.

Interviews at each site were scheduled to fill target quotas for each of the various roles (respondent groups) needed. After each site's chiropractor and facility leaders approved participation in the study, lists of providers by department (or service line) were compiled. Recruitment involved sending individual invitations to VA employees to fill the two-day site visit schedule. Initial invitations were sent by e-mail describing the study and announcing the planned site visit dates, and up to five follow-up contacts were attempted per person. Relatively low subject response rates (45.6%) may have resulted partly from the limited two-day interview timeframe scheduled at each site. Of those who did not participate, 69.5% (*n* = 98) were subjects who did not respond to these initial or follow-up e-mails requests for participation.

#### 6.1.3. Data Collection Instruments

Data collection instruments and protocols in qualitative research are often informal, flexible, and subject to large variations in application. While flexibility represents a strength in traditional qualitative research, it can result in inconsistent and unfocused data collection and variable data quality when qualitative methods are applied in deductive research. For example, interview guides specifying general topics of interest, using broad, open-ended questions, can be very effective in assessing interview subjects' assessment of important concepts and issues and their beliefs and values but ineffective in ensuring that complete and comparable measures of identified variables are collected consistently across a range of subjects (e.g., assessing organizational participants' views or their ratings of concepts or variables deemed important by the research team). In part, the distinction here is between data collection approaches designed to develop new insights and frameworks for understanding and describing the phenomena of interest, versus applying a priori frameworks to collect predefined data and test aspects of these frameworks. Similar problems result from the use of observation guides or protocols lacking adequate specificity and a firm foundation in a priori hypotheses and clearly identified variables: such protocols often produce inconsistent data by (1) encouraging the observer to record events as they unfold and to record a wide range of attributes of the situation under study (whether or not they are deemed relevant to the hypotheses of interest), (2) limiting the likelihood that the observer will note the significance of events that do not occur, and (3) limiting the likelihood that the observer will collect complete, consistent data required for direct comparisons across observation samples.

Considerations of validity, intrusiveness or subject reactivity (Hawthorne effects), and triangulation (to minimize bias) are also too often neglected in deductive applications of qualitative methods. Distinctions between subjective and objective data and between formal and informal organizational structures and processes are also frequently neglected, threatening the validity of study conclusions.

Avoiding these problems requires careful design of data collection plans, based on study goals and hypotheses, involving use of systematic tables or other methods for specifying key variables and suitable, multiple measures. Depending on the importance of each variable and the validity of available measures, two or more data sources are typically needed in qualitative research. Data planning tables listing concepts or variables, definitions, and data sources are effective in ensuring appropriate rigor; data collection instruments (including document coding forms, survey questions, and other data specifications) can be developed directly from these tables.

Rigor and validity are also enhanced through development and use of data collection instrument specifications and training protocols, including variable and measure definitions and instructions in instrument use. When used in research examining health care delivery organizations, such protocols should include plans and instructions for approaching sites, making contacts, arranging interviews/visits, identifying and obtaining documents, following up (to obtain documents and other postvisit/call information), managing informed consent and confidentiality, and so forth. Adequate pilot testing helps ensure the appropriateness of data sources and measures although data collection protocols must be flexible and allow for changes in data collection plans and strategies, when pilot testing fails to reveal valuable new data sources or validity problems that eventually emerge during the main study period.

Finally, study validity is further enhanced through development of data analysis protocols and plans together with the actual instruments, rather than after the completion of data collection. Data planning tables created to guide data collection activities can be used to develop data reporting templates and specifications for translating raw data into variables and preparing for analyses; data from organizational studies are often reported in a standardized “organizational profile” or other comparative formats. These profiles store raw data and summary variables from all data sources, which are then converted into tables for analysis. Additional challenges arise in studies pursuing inductive and deductive study aims simultaneously. For deductive studies with a rigid a priori framework, the number of variables to be measured should be relatively small and easily managed. Inductive, exploratory work involves open-ended questions and unlimited data and is thus more challenging to plan and conduct.

#### 6.1.4. Analyzing Diverse Types of Data

The field of health services research has benefited from several insightful, comprehensive discussions of qualitative research methods and their appropriate use [54]. Proponents have convincingly argued that qualitative methods contribute to findings and insights that cannot be derived from “conventional” or “quantitative” research methods and that research in the clinical, social, and policy sciences requires careful application of both types of approaches to properly study their phenomena of interest.

For qualitative research, patterns of observed individual and organizational practices, behaviors, and outcomes are highly variable (across time and site) and are subject to wide variations and interviewer/observer bias and interpretation. This heterogeneity challenges those designing the research to find and describe consistent patterns and topics within the findings. The patterns and topics identified are heavily influenced by idiosyncratic factors, such as an individual leader's personal views or situation or unrelated pressures or events within a site. Also, each site's situation (planning, implementation, clinic structure, etc.) is influenced by a very large number of factors, whose combined and interacting effects lead to highly variable outcomes (as described in chaos or complexity theory, e.g.). Data collection relying on interviews with individuals entails potential bias, limited validity, and inaccuracies due to challenges in recall and differing perspectives and views of events and differential access to information. Therefore standard challenges in qualitative/case study research also apply here.

Another challenge arises from the limitation that data for many key variables are not always reliably available or are difficult to access, in addition to having questionable validity in some instances. This study also examined a long chain of causal links and multiple determinants and outcomes (independent and dependent variables). Overall, the mixed-methods approach employed here was challenging because dual inductive and deductive research is inherently demanding, for example, deciding how to allocate limited interview time to measure variables identified a priori (for deductive) versus open-ended interviewing to maximize the likelihood of learning something new and interesting (for inductive). Lastly, the lack of available standardized, validated measures, concepts, definitions, and so forth was a significant challenge to this study. These challenges are especially common to pilot studies.

However, there are steps to minimize these biases, including adequate training of data collection staff; comprehensive plans for data collection, validation, and storage; and frequent reviews of data quality and interpretation. While data validity and completeness can be enhanced through the recording of interviews, other methods should also be paired with the traditional recording of interviews such as use of paired interviewers, postinterview debriefing, and other methods. Quality assurance methods should be considered and operationalized for each instrument and data sources. Problems such as incomplete, missing, unusable data should be identified and resolved during the data collection phase rather than after its completion.

Finally, analysis plans are relatively quantitative in most conventional health care studies that often pursue narrow, explicit aims and test explicit, confirmatory hypotheses. However, mixed-methods studies that address both inductive and deductive study questions and employ methods features, such as randomized sampling, that are more typically associated with experiments—combined with open-ended interviews more typically associated with qualitative research—offer what is termed by Morse “alternative forms of evidence” [55, p. 86]. Therefore new opportunities for qualitative inquiries have the ability to emerge [56]. These points are illustrated well by this passage from *Ronald J. Chenail:*

**Take a pragmatic posture to creating studies that marry the most fitting design and methodology choices with the focus of your research curiosity*…* remain true to your interests and then explore a variety of research approaches which can help in the designing and conducting studies to meet your needs. The bottom line is to be pragmatic in creating the design, but remain curious so every reasonable methodological option is considered. However, like taking too many medications can lead to adverse effects to your body, using too many methodologies might produce negative side effects which could be unhealthy for your study. To help remedy this potential risk, please remember this simple research commandment: Thou shall not select an additional methodology for a study, until thou is sure the first methodology selected cannot manage all of the design issues [56].**

## 7. Conclusion 

To our knowledge, VA's introduction of chiropractic services represents the most extensive introduction of any nontraditional medical service into the largest integrated US healthcare system. This is likely to present future research areas of interest to multiple stakeholders. VA policy makers may seek data to inform efforts to best assess and improve the delivery of chiropractic services to meet Veterans' needs. Stakeholders in the chiropractic profession may look to VA's experience as an indicator of future opportunities and integration into other healthcare systems. Other CAM disciplines seeking inclusion into VA and other systems may be interested in the policy and practice implications of VA's chiropractic program. At a broader level, beyond the unique chiropractic and/or CAM implications, our study may have implications for researchers to assess the introduction of any new healthcare service into VA or other large healthcare systems.

For these types of inquiries, research-practice-policy partnerships facilitate research that can be more useful to decision makers (relative to traditional academic research). Decision maker involvement increases the likelihood that (1) useful questions are answered by developing methods and data analyses relevant to service delivery and (2) the interpretation and reporting of results will inform future policy.

Analysis of qualitative observational data in studies combining deductive and inductive aims should be guided by prespecified, model-based hypotheses and detailed analysis plans developed at the outset of the study. Unfortunately, while quantitative analysis methods are well established and accepted, methods for analysis of qualitative data are subject to variability and lack of consensus. Analyses of qualitative data are too often informal, ad hoc, and emergent, with low reliability and validity. These threats can be countered through the use of formal table approaches, in which key variables relevant to each hypothesis are listed in tables and manipulated in a blinded fashion, using qualitative pattern identification and nonparametric quantitative techniques. The analysis tables summarizing and synthesizing information from diverse sources in a standardized format may also serve as reporting tools, in papers and reports. Combining the use of qualitative methods for hypothesis testing and interpretive, inductive applications in this manner represents a powerful application of these methods, using their strengths to enhance management studies and other empirical research in important ways.

In conclusion, qualitative case study research allows for the collection of rich data for deep understanding of the phenomenon of interest. Yet data collection relying on interviews with individuals entails potential bias, limited validity, and inaccuracies due to challenges in recall and differing perspectives and views of events and differential access to information. Also, data for many key variables may be unavailable or difficult to access, in addition to having questionable validity in some instances. Researchers who are not trained in qualitative methods (and who are accustomed to conducting empirical research using quantitative methods alone) are less likely to be interested in applying qualitative methods in inductive or interpretive research but can—and should—be interested in applying qualitative methods to enhance the data available for more conventional deductive forms of empirical research. The use of mixed methodology can enrich health services research testing a priori study hypotheses and narrowly defined research questions and can also help suggest detailed causal explanations and generate important new exploratory questions and findings as well.

## Figures and Tables

**Table 1:** Key domains and variables.

Domains	Variables
Environment and context	Societal, VA, VISN (region), and facility characteristics
Clinic planning and implementation	Planning process, milestones, communication and coordination, resources, and team characteristics
Clinic structure	Clinic characteristics and staff characteristics and competencies
Care processes	Clinical services provided and quality of care
Impacts and outcomes	Quality of implementation, satisfaction with processes, services, and care (patient, leadership, and staff)
